# Propolis-loaded liposomes: characterization and evaluation of the *in vitro* bioaccessibility of phenolic compounds

**DOI:** 10.5599/admet.2204

**Published:** 2024-02-05

**Authors:** Oznur Saroglu, Ayse Karadag

**Affiliations:** Food Engineering Department, Chemical and Metallurgical Engineering Faculty, Yildiz Technical University, Istanbul, Turkey

**Keywords:** Ammonium phosphatides, Tween 80, in vitro digestion, encapsulation, flavonoids

## Abstract

**Background and purpose:**

Propolis has low water solubility, poor stability, and limited bioaccessibility of phenolic constituents when subjected to *in vitro* digestion. To overcome these drawbacks, the liposomal encapsulation method can be employed.

**Experimental approach:**

Soybean phosphatidylcholine lecithin mixed with Tween 80 (T80) and ammonium phosphatides (AMP) was used to produce propolis extract (PE)-loaded liposomes. The mean particle size, zeta potential, encapsulation efficiency values, and transmission electron microscopy analysis were used to characterize liposomes. Individual phenolics were determined for digested and nondigested propolis-loaded liposomes and propolis extract.

**Key results:**

Tween 80 incorporation reduced the size of unloaded liposomes, whereas AMP inclusion yielded larger liposomes. In both formulations, PE loading significantly increased the size and reduced the zeta potential values and homogeneity of the size distribution. In free PE, the most bioaccessible polyphenols were phenolic acids (3.20 to 5.63 %), and flavonoids such as caffeic acid phenethyl ester, galangin, pinobanksin, and pinocembrin (0.03 to 2.12 %) were the least bioaccessible. Both liposomal propolis provided significantly higher bioaccessibility of phenolic compounds. The liposomes with T80 and AMP in their compositions recovered 52.43 and 185.90 % of the total amount of phenolic compounds in the nondigested samples, respectively. The liposomes containing AMP not only exhibited high solubility for PE but also provided protection to the phenolic compounds during *in vitro* digestion.

**Conclusion:**

Liposomal encapsulation could be a promising approach to improving the solubility and stability of PE in digestive fluids, making it suitable for the delivery of propolis in oral formulations.

## Introduction

Propolis, a natural resinous beehive product, exhibits diverse biological and pharmacological activities due to the presence of flavonoids, phenolic acids, terpenes, sesquiterpene-alcohols, and their derivatives [1]. Specifically, among flavonoids, the caffeic acid phenyl ester (CAPE), chrysin, galangin, apigenin, quercetin, kaempferol, naringenin, rutin, taxifolin, pinobanksin, and pinocembrin have been reported as the major active components of propolis [2].

Although propolis is commercially available in different formulations such as capsules, powders, sprays, and drops, its acceptance by consumers and wider application in food products are limited due to its low water solubility, poor stability, strong and unpleasant taste, and odor. Additionally, the low bioaccessibility of phenolic compounds in propolis upon digestion has been reported previously [3-5]. The bioaccessibility of a phytochemical is the fraction released from the food matrix during digestion in a form accessible for absorption in the small intestine or biotransformed by the gut microbiota. It is impacted by many factors, including the nature of the food matrix, the nature of the phytochemical, and the processes occurring inside the gastrointestinal tract [6]. To improve the bioaccessibility of propolis, different encapsulation methods have been employed, such as spray-drying and spray-chilling [7], complex-coacervation [8], polymeric nanoparticles [9,10], multiple-emulsions [11], liposomes [12-14], and hybrid systems such as liposomal propolis-loaded nanofibers [15].

Liposomes are spherical bilayer vesicles formed from aqueous dispersions of phospholipids, with typical sources of phospholipids being soy or egg lecithins [16]. Modifications to the traditional liposome composition, such as incorporating phospholipids with other surface active materials, such as polyethylene glycol [17], bile salts [18], and Tween 80 [19], yielded higher loading of hydrophobic compounds with elevated cellular uptake of active compounds [20].

Ammonium phosphatides (AMP), an authorized food additive (E 442) in the EU, is used as an emulsifier to be an alternative to lecithin in the form of ammonium-neutralized phosphoric esters of mono- and diglycerides. They consist of a mixture of phosphatidic acids, where the fatty acid composition depends on the source of vegetable oil. Apart from commercial use in chocolate, there have been a few other applications, such as improving the volume of white bread, enhancing the texture of chewing gum, and retarding the oxidation of vegetable oils [21]. The latest scientific opinion of the EFSA Panel on Food Additives and Nutrient Sources added to Food (ANS) re-confirmed its acceptable daily intake (ADI) of 30 mg/kg body weight (bw) per day. For example, within the EU, the use of AMP is permitted at 10,000 mg/kg in chocolate-based products [22], and its application at the *quantum satis* level is allowed as a carrier for use in food antioxidants [23].

The propolis loading only ranged from 0.25 to 0.5 % (w/v) in the previous studies [12-14] related to propolis-loaded liposomes. In our previous study [15], the highest propolis loading in liposomes produced without the aid of any surfactant was 0.6 % (w/v); higher than this concentration resulted in the precipitation of propolis. In this study, we aimed to enhance the loading of propolis into liposomes at higher concentrations (2 and 4 %, w/v) with the use of two different surfactants. There were some studies about the *in vitro* digestion of propolis extracts collected from different regions [4,24], prepared by different solvents [5,25], and propolis encapsulated in spray-dried particles [7,26], in freeze-dried gum Arabic [27], in cyclodextrin particles [28], and in starch nanoparticles [29].

Therefore, the present study aimed to prepare propolis-loaded liposomes and investigate the *in vitro* bioaccessibility of the phenolic compounds of propolis. Two different liposomal propolis formulations were prepared by incorporating either Tween 80 or AMP. To the best of our knowledge, AMP has been employed for the first time in the production of liposomes. Propolis extract and two different liposomal propolis were subjected to a simulated *in vitro* digestion model, and the change of phenolic compounds at the gastric and intestinal stages of digestion was evaluated by LC-MS/MS analysis. In addition to the evaluation of their morphology by transmission electron microscopy (TEM) analysis, the propolis-loaded liposomes were characterized in terms of particle size, zeta potential, polydispersity index, and encapsulation efficiency of propolis phenolics.

## Experimental

### Materials

Lipoid S75 (70 % phosphatidylcholine) was purchased from Lipoid (Ludwigshafen, Germany). Ethanol was purchased from Tekkim (Istanbul, Turkey). Amylase (A1031), pepsin (P7012), pancreatin (P7545), bile (B3883), polyoxyethylene sorbitan monooleate (Tween 80), Amicon® membrane ultra-centrifugal filter (50 kDa), and analytical standards were bought from Sigma-Aldrich Ltd (Steinheim, Germany). AMP 4455 (Ammonium Phosphatide) was kindly provided by Palsgaard A/S (Istanbul, Turkey). Ethanolic extract of propolis (PE) was donated from Balparmak (Altıparmak Gıda, Istanbul, Turkey).

### Preparation of propolis extract-loaded liposomes

The liposome was prepared using the thin-film hydration method, according to the method of Saroglu *et al.* [30], with some modifications. Soy lecithin (Lipoid S75), Tween 80 (T80), ammonium phosphatide (AMP), and propolis extract (PE) were dissolved in ethanol. The mass ratio was decided based on preliminary tests and our previous study [15]. Our goal was to use the minimum surfactant concentration that would allow us to load the highest PE in the final liposome without precipitating. For this purpose, we tried different lecithin to surfactant (AMP or T80) concentrations ranging from 16:1 to 4:1 (w/w), and continued with 5:1 (w/w), in which for both surfactants, there was no precipitation of PE in the final liposomes. The solvent was evaporated using a rotary evaporator (Buchi-R-210, Essen, Germany) under vacuum (40 °C, 70 mmHg). The dried film layer was suspended in distilled water, homogenized by an Ultra-Turrax (IKA T-18, Staufen, Germany) for 5 min at 10,000 rpm, and sonicated (0.5 cycles, 60 % amplitude) for 7 min with an ultrasonic processor (UP400S, Hielscher, Berlin, Germany). The final concentration of lecithin in the liposomal dispersions was 4 % (w/v), and PE loading was 2 and 4 % (w/v).

### Mean particle size and zeta potential measurements

The z-average particle diameter and zeta (*ζ*) potential of liposomes were analyzed by using a dynamic light scattering (DLS) instrument (Nano ZS, Malvern, Worcestershire, UK). The refractive index values of lecithin and water were taken as 1.44 and 1.33, respectively. All samples were diluted to 1000 (v/v) fold with distilled water to avoid multiple scattering effects, and measurements were made on freshly prepared liposomes at 25 °C. The results were given as the mean ± standard deviation of nine measurements [31].

### Transmission electron microscopy analysis

Transmission electron microscopy (TEM) analysis of liposomes was conducted with the negative staining technique [32]. After diluting with water, drops of suspensions were placed onto copper grids (200 mesh), allowing them to settle and fix. Then, the grids were negatively stained with a 2 % (w/v) aqueous solution of uranyl acetate and dried at room temperature. The grids were analyzed by TEM (Hitachi HT7800, Tokyo, Japan) operating at a 100 kV acceleration voltage.

### Encapsulation efficiency of PE in liposomes

To determine the encapsulation efficiency (EE), free PE was separated from the PE-loaded liposomes by centrifuging at 4000*g* for 40 min at 4 °C using an Amicon membrane ultra-centrifugal filter (50 kDa) [15]. 1 mL of liposome was added to the upper section of the filter, and free PE was filtered to permeate (lower section), while the PE-loaded liposomes stayed in the retentate phase (upper section). The amount of free PE in permeate could be lower for detection with the spectrophotometric assay; therefore, both phases were measured to determine the EE of PE. The retentate phase was mixed with ethanol and centrifuged at 4000*g* for 30 min at 4 °C to precipitate the lecithin, and the supernatant was used for PE determination. The unloaded liposome (with no PE) was also processed similarly as a blank for spectrophotometric assays. The calculation was given based on the free PE filtered to permeate, but the values were also verified by the calculation of PE recovered from the retentate of the Amicon filter. The amount of PE was calculated based on the total phenolic content (TPC) determined by the Folin-Ciocalteu (FC) method [33]. EE was estimated using equation (1).



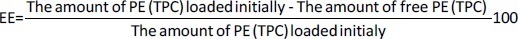

(1)


### Analysis of phenolic compounds of PE by LC-MS/MS

LC-MS/MS analysis was performed according to the method of Guzelmeric *et al.* [2]. Identification of polyphenol was performed with a Waters Acquity UPLC H-class system coupled to a Waters Xevo TQD triple quadrupole mass spectrometer (Waters Corporation, Milford, MA USA) equipped with an electrospray ionization (ESI) source. For chromatographic separation, the Cortecs T3 (Waters Corporation, Milford, USA) column (1.6 μm particle size, 2.1×150 mm) was used. All samples passed through 0.22 μm membrane filters, and the injection volume was 5 μL. Gradient program with two solvents (mobile phase A; the acetic acid/MQ water (1/10000 v/v), mobile phase B; 80:20 acetonitrile: methanol (v/v) at a gradient flow rate of 0.25 μL/min). The gradient elution was as follows: 2 % B (0-1.30 min), 2 to 55 % B (1.30-35 min), 55 to 95 % B (35 to 37 min), 95 to 2 % B (37-37.01 min), and 2 % B (37.01-40 min). The autosampler and column oven temperatures were maintained at 10 °C and 30 °C, respectively. The parameters of ESI-MS/MS were adjusted as follows: the ion source and desolvation temperature were set at 150 and 450 °C, respectively. The capillary voltage was set at 2 kV. Desolvation and cone gas flows were 850 and 50 L/h, respectively. LC-MS/MS data was processed using Waters® Mass-Lynx software at Target Lynx Program (Waters).

### In vitro simulated digestion analysis

**Figure 1.**
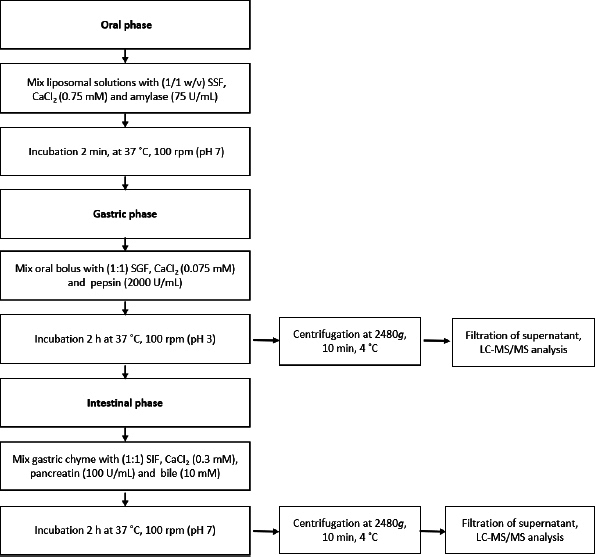
The flowchart of In vitro simulated digestion analysis

The in vitro simulated digestion assay was conducted according to the method of Brodkorb et al. [34] and Minekus et al. [35] (Figure 1).

The samples were mixed in a 1/1 ratio (w/v) with simulated salivary fluid (SSF), α-amylase (75 U/mL), and CaCl_2_ (0.75 mM), and then vortexed for 2 min at 37 °C (pH 7.0). The oral bolus was diluted with simulated gastric fluid (SGF) (1/1, v/v), containing CaCl_2_ (0.075 mM) and pepsin (2000 U/mL), and incubated for 2 h at 37 °C, 100 rpm (pH 3.0). The gastric chyme was diluted with simulated intestinal fluid (SIF) (1/1, v/v), CaCl_2_ (0.3 mM), pancreatin (100 U/mL), and fresh bile (10 mM), and incubated for 2 h at 37 °C, 100 rpm (pH 7.0). The test tubes taken at each digestion step were centrifuged at 2480*g*, 10 min at 4 °C, and filtered supernatants (0.45 μm) were immediately frozen in liquid nitrogen and lyophilized. A blank test tube without a sample but with all digestion fluids was also subjected to analysis. The lyophilized supernatants of the oral, gastric, and intestinal phases were dissolved in 80 % aqueous methanol acidified with 0.1 % HCl (v/v). All procedures were done in triplicate. The bioaccessibility index (BI) was determined with the following equation.



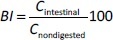

(2)


where *C*_intestinal_ is the compound concentration in the intestinal phase, *C*_nondigested_ is the initial concentration of the compound in the nondigested sample.

### Statistical analysis

All experiments were carried out in triplicate, and the data were reported as the mean ± standard deviation. Statistical analysis was performed with SPSS Statistics Software (IBM version 20, Armonk, NY, USA USA). Significant differences between means (*p*<0.05) were assessed by one-way analysis of variance (ANOVA) followed by Tukey’s post hoc test. The mean values of the phenolic composition of L1-PE4 and L1-PE2 samples were analyzed by an independent-sample *t*-test. Using Origin Pro 2023 statistical analysis software (Origin Lab Corp., MA, USA), a heat map was created to more effectively display the data (the variation of various phenolic compounds at each digestion stage and BI values for each sample) [36].

## Results and discussion

### Particle size, zeta potential and morphology of PE-loaded liposomes

**Figure 2.**
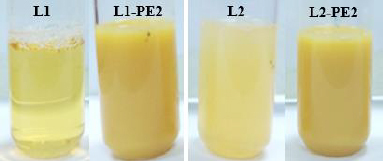
L1 and L1-PE2 were unloaded and PE (2 %, w/v) loaded liposomes incorporated with T80, respecttively. L2 and L2-PE2 were unloaded and PE (2 %, w/v) loaded liposomes incorporated with AMP, respectively.

The photographs of unloaded liposomes prepared by the incorporation of T80 (L1) or AMP (L2) and their PE-loaded (2 %, w/v) counterparts (L1-PE2 and L2-PE2) are given in Figure 2. Our previous study determined that PE loading into the liposomes prepared without surfactant was limited. The highest PE loading was achieved at 0.6 % (w/v), and the incorporation of Tween 80 (T80) enabled us to load PE at higher concentrations (≥ 2 %) [15]. Therefore, in this study, in addition to T80, we employed AMP in the liposome formulations. Both T80 and AMP were permitted food additives to be used as surfactants. The combination of phospholipids with other surfactants provided advantages, such as increasing the solubility of the active substance and loading capacity. It has also been suggested that other surfactants could alter the liposomal membrane structure, potentially creating more space for the active substance to be embedded during encapsulation [37-40].

We were able to load PE in liposomes prepared by the inclusion of T80 (L1) at two concentrations of 2 and 4 % (w/v) (L1-PE2 and L1-PE4), whereas, in liposomes with AMP, we were able to load only 2 % PE (w/v) (L2-PE2), and with higher loading, PE was precipitated. The encapsulation efficiency of PE phenolics in liposome formulations was 70.27±1.13 % in the L2 formulation, and at the same PE loading, it was 75.49±1.44 % in the L1 formulation and reduced to 68.01±6.05 % (*p*>0.05) at a higher level of PE loading (4 %, w/v) (Table 1). Our EE values of PE in liposomes were comparable to the values (35-82 %) of the phosphatidylcholine liposomes prepared previously to encapsulate PE [12-14].

**Table 1.** Encapsulation efficiency, mean particle size, zeta potential, and polydispersity index (PDI) values of liposome samples.

Liposome	Surfactant	EE, %	Average particle size, nm	Zeta potential, mV	PDI
L0	-	-	64.61±16.43	-39.23±1.15	0.28±0.09
L1	T80	-	47.18±1.31^b^	-37.00±1.37^b^	0.36±0.02^c^
L1-PE2		75.49±1.44^a^	103.41±4.09^b^	-34.33±2.02^ab^	0.46±0.02^b^
L1-PE4		68.01±6.05^a^	370.47±43.21^a^	-30.93±1.37^a^	0.54±0.04^a^
L2	AMP	-	80.97±0.99^b^	-36.53±0.76^b^	0.44±0.01^a^
L2-PE2		70.27±1.13	179.30±2.90^a^	-33.80±1.28^a^	0.36±0.01^b^

The values with different superscript lowercase letters within the same liposome group (L1 or L2) are significantly different (*p*<0.05). L0 was the unloaded liposome formulation without added surfactant. L1 and L1-PE2 were unloaded and PE (2 %, w/v) loaded liposomes incorporated with T80, respectively. L2 and L2-PE2 were unloaded and PE (2 %, w/v) loaded liposomes incorporated with AMP, respectively. PE: propolis extract; T80: Tween 80, polyoxyethylene sorbitan monooleate; AMP: ammonium phosphatide 4455.

The particle size of unloaded liposomes (without PE) was 47.18±1.31 nm when T80 was incorporated (L1), and it was 80.97±0.99 nm when AMP was included (L2) in the preparation of liposomes (Table 1). When we prepared the unloaded liposomes without any synthetic emulsifier, the size was 64.61±16.43 nm, so the interaction of T80 and AMP with the phospholipid bilayer could be different. Incorporating an amphiphile with a large head group, as in T80, could induce high curvature of the liposomal membranes [41]. Therefore, the reduced particle size of liposomes by T80 incorporation could be due to a steric repulsion among the T80 surfactants, which are exposed from the outer and inner leaflets of the liposomal bilayer membrane. The T80 surfactants exposed to the outer leaflet of the bilayer membrane increased the liposome particle curvature, whereas the T80 exposed to the inner leaflet did the opposite. Therefore, incorporating T80 surfactant reduced the liposomal size since more T80 existed in the outer leaflet than in the inner leaflet of the liposomal bilayer membranes [42]. AMP could be more associated with the liposomal surface, increasing the thickness. It could be possible that the ammonium moiety of the phosphatide, due to its stronger basicity [21], exhibits a higher affinity for the liposomal surfaces. The zeta potential values of liposomes prepared without synthetic surfactants (AMP or T80) were -39.23±1.15 mV and slightly decreased by the incorporation of T80 (-37.00±1.37 mV) or AMP (-36.53±0.76 mV), which could support our findings with particle size measurements that both surfactants were more related to the surface of soy lecithin liposomes.

Compared to unloaded liposomes, loading PE in both formulations increased the mean particle size and reduced the zeta potential values significantly (*p*<0.05) (Table 1). Considering the hydrophobic properties of PE, it was expected to be more included in the bilayers than in the internal aqueous phase of the liposome. Therefore, higher loading of PE in liposomes would result in the formation of larger liposomal vesicles [12,13,15]. The reduced zeta potential values might also indicate that PE was associated with the surfaces. Many studies have shown that phenolic compounds interact with liposome surfaces through both hydrophilic and hydrophobic interactions [13,43]. The low polydispersity index (PDI) value is a measure of the size-based heterogeneity of a given sample. PE loading at each concentration decreased the homogeneity of our liposomal dispersions containing T80. However, the particle size distribution of liposomes containing AMP became more homogenous when loaded with PE.

**Figure 3.**
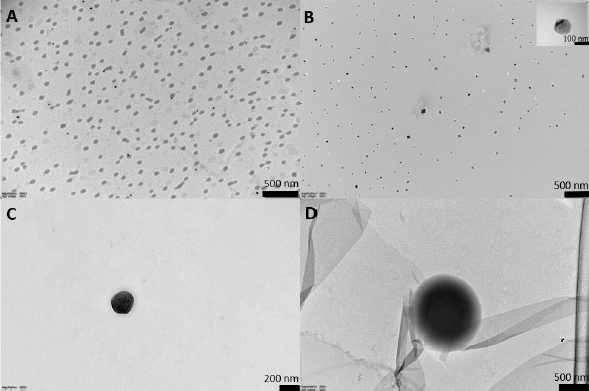
TEM images of L1 (A), L1-PE2 (B), L2 (C) and L2-PE2 (D) liposomes. L1 and L1-PE2 were unloaded and PE (2 %, w/v) loaded liposomes incorporated with T80, respectively. L2 and L2-PE2 were unloaded and PE (2 %, w/v) loaded liposomes incorporated with AMP, respectively. The scale bar in A, B and D was 500 nm, and it was 200 nm in C. The scale bar of the inner picture in B was 100 nm.

The liposome morphology was examined using TEM. The cation stain with uranyl ion that binds with the phosphate group of phospholipids allowed the visualization of liposome structures with a near-spherical or spherical morphology in formulations (Figure 3). PE loading and AMP enhanced the staining affinity of the liposomes, and the vesicles got darker. Compared to the size distribution of unloaded liposomes observed in Figure 3A, PE loading reduced the homogeneity of the size distribution in Figure 3B. While some vesicles decreased in size, others increased.

### In vitro simulated digestion

The individual phenolic compounds and their concentrations in the PE are given in Table 2. The phenolic profile of PE was typical for poplar propolis with the presence of cinnamic acid derivatives (*p*-coumaric acid, caffeic acid, *trans*-cinnamic acid, *trans*-ferulic acid, and 3,4-dimethoxycinnamic acid), including CAPE, several flavonols (quercetin, kaempferol, galangin, and pinobanksin), flavones (luteolin, apigenin, chrysin), and flavanones (pinocembrin, naringenin). The main phenolic compounds in PE were pinocembrin (18.64±0.54 mg/g), galangin (14.36±1.48 mg/g), pinobanksin (14.06±0.98 mg/g), and CAPE (12.66±0.74 mg/g) which were also reported in European propolis samples [2,4,44].

**Table 2.** Phenolic composition of PE and PE-loaded liposomes by LC-MS/MS method, ionization mode ESI(-)

Compounds	Molecular formula	RT, min	Precursor ion *m*/*z*	Product ion(s) *m*/*z*	Cone voltage, V	Collision energy, eV	PE content, mg/g	Content, μg/mL liposome	
								L1-PE4	L2-PE2
3,4 dimethoxy cinnamic acid	C_11_H_12_O_4_	19.1	206.7	102.7	25	20	6.62±0.47	440.88±8.64^a^	207.72±4.07^b^
Apigenin	C_15_H_10_O_5_	25.0	269	117.3/149/151	40	30/25/25	3.30±0.14	187.22±6.52^a^	88.21±3.07^b^
Caffeic acid	C_9_H_8_O_4_	10.9	179	135	25	20	4.19±1.15	385.78±11.69^a^	181.76±5.50^b^
CAPE	C_17_H_16_O_4_	32.4	283	179/161/135	25	20	12.66±0.74	649.48±1.41^a^	306.01±0.66^b^
Chrysin	C_15_H_10_O_4_	30.9	253	225/209/151	25	20	9.31±1.08	450.72±11.54^a^	212.36±5.44^b^
Trans-ferulic acid	C_10_H_10_O_4_	14.7	193	178/149/134	25	20	1.60±0.21	131.58±8.66^a^	61.99±4.08^b^
Galangin	C_15_H_10_O_5_	31.9	269	197/213/227	25	20	14.36±1.48	824.20±3.25^a^	388.33±1.53^b^
Kaempferol	C_15_H_10_O_6_	25.4	285	93/151/257	25	20	3.68±0.15	208.97±24.57^a^	98.45±11.57^b^
Luteolin	C_15_H_10_O_6_	22.3	285	133/241/267	25	20	0.94±0.02	61.80±10.78^a^	29.12±5.08^b^
Naringenin	C_15_H_12_O_5_	24.2	271	145/151	25	20	2.07±0.06	106.18±8.01^a^	50.03±3.78^b^
p-coumaric acid	C_9_H_8_O_3_	13.5	163	93/119/147	25	20	2.14±0.27	191.21±3.92^a^	90.09±1.85^b^
Pinobanksin	C_15_H_12_O_5_	24.3	271.2	153/225/253	25	20	14.06±0.98	616.90±34.34^a^	290.66±16.18^b^
Pinocembrin	C_15_H_12_O_4_	31.1	255	151/171/213	25	20	18.64±0.54	1291.69±3.12^a^	608.58±1.47^b^
Quercetin	C_15_H_10_O_7_	22.4	301	150.8/178.9	35	20	1.67±0.18	151.02±4.29^a^	71.15±2.02^b^
Trans-cinnamic acid	C_9_H_8_O_2_	21.1	147	77/102.8	25	20	2.33±0.12	183.47±28.99^a^	86.44±13.66^b^
**Total**							**97.56**	**6012.19**	**2782.96**

Data are expressed as mean ± S.D. of triplicate measurements. The values with different superscript lowercase letters between L1-PE4 and L2-PE2 samples are significantly different (*p*<0.05). RT: retention time. PE: propolis extract; L1-PE4: PE (4 %, w/v) loaded liposomes incorporated with T80; L2-PE2 : PE (2 %, w/v) loaded liposomes incorporated with AMP. T80: Tween 80, polyoxyethylene sorbitan monooleate; AMP: ammonium phosphatide 4455.

PE and PE-loaded liposomes (L1-PE4 and L2-PE2) were subjected to an in vitro digestion procedure, and the change of phenolic compounds at the gastric and small intestinal stages was analyzed using LC-MS/MS, and the results were given in Table 3. The in vitro digestion procedure was applied to the PE without dissolving it in alcohol or any other solvent prior to digestion. Between two L1-PE formulations, the assay was conducted with the formulations that allowed higher PE loading. We omitted the analysis of the oral stage due to the liquid nature of liposomal propolis and the short duration of the digestion stage.

**Table 3.** The change of phenolic compounds of PE and PE-loaded liposomes during *in vitro* simulated digestion

Compounds	PE content, μg/g		BI, %	L1-PE4 content, μg/mL liposome		BI, %	L2-PE2 content, μg/mL liposome		BI, %
	SGF	SIF		SGF	SIF		SGF	SIF	
3,4 dimethoxy cinnamic acid	153.85±13.15^b^	367.82±66.87^a^	5.63	193.67± 9.60^b^	747.00±6.00^a^	169.46	88.66±16.89^b^	586.23±25.95^a^	282.28
Apigenin	-	-	-	-	36.33±4.51^a^	19.38	-	90.60±10.32^a^	102.71
Caffeic acid	196.22±6.85^a^	122.94±22.52^b^	3.20	206.33±12.0^a^	193.33±2.52^a^	50.16	82.33±8.81^b^	176.37±2.57^a^	97.03
CAPE	-	3.56±1.19^a^	0.03	1.00±0.00^b^	130.33±33.50^a^	20.07	-	205.00±34.64^a^	66.99
Chrysin	-	56.78±11.75^a^	0.61	2.33±0.58^b^	322.00±51.00^a^	71.32	1.92±0.08^b^	644.90±76.82^s^	303.68
*Trans*-ferulic acid	60.35±7.63^a^	41.68±0.00^b^	2.64	96.67±6.11 ^b^	167.33±40.50^a^	127.23	37.96±8.885^b^	149.60±40.21^a^	241.33
Galangin	54.96±18.32^a^	84.20±17.34^a^	0.60	8.00±1.00^b^	40.00±3.00^a^	4.85	9.24±2.13^b^	81.80±0.69^a^	21.06
Kaempferol	-	77.48±22.10^a^	2.09	-	10.33±0.58^a^	4.98	-	6.40±0.46^a^	6.50
Luteolin	35.87±11.96^a^	29.58±9.86^a^	3.13	5.33±1.53^b^	15.00±0.00^a^	24.73	7.69±1.66^b^	52.00±13.89^a^	178.57
Naringenin	4.54±1.05^b^	45.38±4.96^a^	2.19	8.67±1.53^b^	114.00±18.00^a^	106.92	7.81±1.22^b^	205.40±5.24^a^	410.55
*p*-coumaric acid	62.80±6.29^b^	103.36±20.56^a^	4.96	77.67±6.81^b^	190.00±16.00^a^	99.44	43.72±1.37^b^	285.90±44.77^a^	317.35
Pinobanksin	81.54±11.05^b^	298.60±28.95^a^	2.12	72.67±2.31^b^	554.00±42.00^a^	90.20	61.04±4.29^b^	1440.70±105.01^a^	495.67
Pinocembrin	21.96±0.42^b^	292.59±23.50^a^	1.57	14.33±2.08^b^	509.00±99.00^a^	39.41	9.34±0.69^b^	990.80±107.59^a^	162.81
Quercetin	54.41±11.61^a^	34.82±7.61^a^	2.05	3.67±0.58^b^	12.67±0.58^a^	8.39	5.94±1.22^b^	41.00±5.00^a^	57.62
Trans-cinnamic acid	52.24±17.41^b^	117.76±3.36^a^	5.06	29.3±7.09^b^	112.00±13.00^a^	61.31	9.16±1.16^b^	209.25±16.65^a^	242.08
**Total**	**778.73**	**1676.55**	**1.72**	**719.33**	**3153.00**	**52.43**	**364.92**	**5173.55**	**185.90**

Data are expressed as mean ± S.D. of triplicate measurements. The values with different superscript lowercase letters between digestion steps for each sample are significantly different (*p*<0.05). BI: Bioaccessibility index, SGF: Simulated gastric fluid; SIF: simulated intestinal fluid; PE: propolis extract; L1-PE4: PE (4 %, w/v) loaded liposomes incorporated with T80; L2-PE2 : PE (2 %, w/v) loaded liposomes incorporated with AMP. T80: Tween 80, polyoxyethylene sorbitan monooleate; AMP: ammonium phosphatide 4455.

Due to the low solubility of PE in water, there have been many attempts to find alternative oral delivery systems employing safe solvents and additives for human consumption without compromising its bioactive properties.

It was previously reported that the low pH and pepsin enzyme in the gastric stage of digestion had a slight effect on the liposomal membranes, whereas together with bile salts and pancreatic enzymes that also have lipolytic activity, they can damage the structure of liposomes and enhance the release of encapsulated compound [31,45]. The liposomal delivery system increased the aqueous solubility of PE in both stages of digestion. In the gastric stage, the phenolic compounds of PE detected at the highest level were 3,4-dimethoxy cinnamic acid and caffeic acid in all samples (Table 3). In the gastric fluids, pinobanksin was detected at a higher concentration compared to pinocembrin, despite pinocembrin being present in higher quantities in the initial non-digested PE. The additional hydroxyl group of pinobanksin may provide a higher solubility in aqueous solutions. In the study of Sun *et al.* [46], who compared the phenolic profiles of propolis extracts prepared by a mixture of ethanol and water, the change in pinobanksin content in samples was less prominent with increasing alcohol concentration compared to the content of pinocembrin. Compared to the gastric stage, the concentrations of most phenolics were elevated by the following intestinal stage. Ozdal *et al.* [4] also reported an increase in total phenolic and flavonoid contents after the intestinal phase, when ethanol extracts of 11 different Turkish propolis extracts were subjected to digestion.

At the intestinal stage of digestion, PE showed a reduction in the concentrations of caffeic acid, *trans*-ferulic acid, and quercetin while the concentrations of the other phenolic compounds increased. Meanwhile, in liposomal PE, the concentration of all phenolic compounds increased (Table 3). It was also reported that the existence of the C3-OH group in the C-ring, the catechol moiety in the B-ring, and the C2=C3 bond in the C-ring play important roles in the stability of flavonoids, including in alkali conditions [47] of intestinal fluid. Quercetin has two OH groups on its B-ring compared to galangin (no -OH) and kaempferol (one -OH), therefore, it could have been more susceptible to alkali intestinal fluids. For example, Alvarez-Diduk *et al*. [48] incubated kaempferol and quercetin at high pH conditions (pH > 9), and kaempferol was more stable in alkali buffer. The higher concentration of phenolic compounds in intestinal fluids compared to the gastric stage could be related to increased solubility of phenolics due to the additional incubation time, higher pH value, and the presence of enzymes and bile salts that could act on the resinous residues of PE. PE phenolics were generally reported to have a weak acidic character, their solubility increases at pH values higher than their p*K*a values. For example, p*K*_a_ values for caffeic acid were 4.43 and 8.69 [49], for kaempferol 7.05, 8.88, and 9.92, for quercetin 5.87, 7.12, and 8.43 [50], and for galangin 6.8, and 9.4 [51] making them more soluble at higher pH values.

We also evaluated the bioaccessibility index (BI) values of phenolic compounds in PE by comparing the amount determined at the end of the digestion (Table 3) to the initial non-digested samples (Table 2). The BI was very low in PE when it was subjected to *in vitro* digestion as it was. The highest recovery was determined for 3,4 dimethoxy cinnamic acid (5.6 %), *p*-coumaric acid (4.9 %), and *trans*-cinnamic acid (5.1 %), and it was very low for CAPE (<0.1 %), galangin (0.6 %), pinobanksin (2.1 %), and pinocembrin (1.5 %). Whereas when PE was delivered by a liposomal system to *in vitro* digestion, high levels of BI values were calculated. The concentration of phenolic compounds at the end of intestinal digestion was always higher in liposomal propolis that incorporated AMP into its structure (L2-PE2). The highest BI was achieved for 3,4 dimethoxy cinnamic acid (169.4 %), followed by *trans*-ferulic acid (127.2 %) in the L1-PE4 liposome. In the L2-PE2 liposome, among phenolic acids, the highest BI was achieved for *p*-coumaric acid (317.4 %), followed by 3,4 dimethoxy cinnamic, *trans*-ferulic, and *trans*-cinnamic acids.

**Figure 4.**
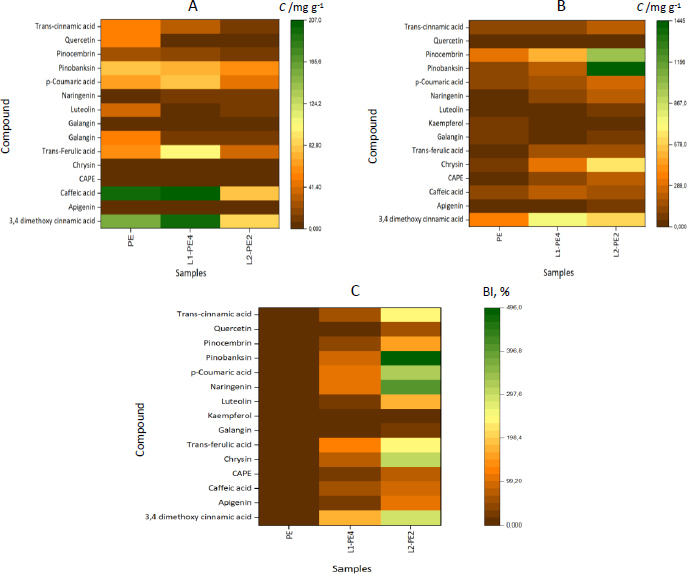
Heat maps representing the change of concentrations of individual phenolic compounds in the gastric (A) and intestinal (B) stages of *in vitro* digestion, and BI values (C)

We created a heat map for visual representation (Figure 4) and interpretation of the data.

The more intense the color, the greater the intensity of the individual phenolics released from propolis-loaded liposomes (L1PE4 and L2-PE2) and propolis extract at each stage of *in vitro* digestion. When the individual phenolics and BI values were low, the colors were more brown and converted to the orange-yellow at higher values. For example, in the gastric stage, L2-PE2 liposomes have been surpassed by brown, dark yellow, and orange colors, whereas L1P4 and PE have both green and light yellow color strengths. At the following intestinal digestion stage, on the other hand, the heat map image of PE was almost covered in brown, whereas L2-PE2 had light yellow and green tints, indicating that more of those individual phenolics could be recovered from digestion fluids. As expected, the BI values of individual phenolics were very low (dark brown) for free PE, and the highest BI values for almost every phenolic compound were attained for L2-PE2 liposomes, except for kaempferol, which was similar to those of the other two samples.

In a recent study, when PE extracts solubilized in ethanol and lactic acid were analyzed after *in vitro* simulated digestion steps, most of the phenolic compounds, including quercetin, kaempferol, apigenin, and luteolin, could not be detected at the intestinal stage, with the recovery of pinocembrin and *trans*-cinnamic acid at 43 to 50 % and 62 to 74 %, respectively [5].

In terms of phenolic compounds specific to propolis, the BI of pinobanksin (90.2 %), pinocembrin (39.4 %), CAPE (20.1 %), and apigenin (19.4 %) was higher than those of galangin (4.85 %), kaempferol (4.98 %), and quercetin (8.39 %) in the L1-PE4 liposome. The total recovery of phenolics was 52.43 % of the initial concentration in the L1-PE4 liposome. Although, compared to PE, the L1 liposome provided enhanced solubility to the phenolic compounds of PE in the digestion medium, the concentration of the phenolic compounds was mostly decreased by digestion. Compared to the initial non-digested L2-PE2 liposome, the sample after digestion showed 1.62- and 4.95-times higher amounts of pinocembrin and pinobanksin concentration, and the other phenolic compounds specific to PE were also mostly retained after digestion. For example, BI for apigenin and CAPE were 103 and 67 %, respectively. The lowest recovery was determined for kaempferol (6.50 %) and galangin (21.06 %), and the total recovery of phenolics in the L2-PE2 liposome was 185.90 % of the initial concentration. Therefore, it could be concluded that the L2-liposomal formulation increased PE's solubility and provided protection to phenolic compounds during digestion.

The protection of liposomal encapsulation of phenolic compounds through digestion was previously reported. The well-organized assembly of phospholipids in liposomes could be protected from membrane degradation during gastric digestion [31]. The reasons for the enhanced release of encapsulated phenolics during the intestinal digestion of liposomal propolis could be associated with the swelling of liposome vesicles, increased membrane fluidity due to the permeation of bile salts to the phospholipid membrane, and the enzymatic hydrolysis of phospholipid structure [52]. The modification of the membrane layer provided by the incorporation of AMP could better protect the PE phenolics compared to the liposomes containing T80 in the structure. The retention of encapsulated compounds by the liposomal encapsulation system in digestive fluids would also depend on the bioactive compound. For example, when the cocoa procyanidin-rich phenolic extract was encapsulated in liposomes, compared to their nonencapsulated counterparts, the bioaccessibility increased for all catechins but not alkaloids [53]. Hu *et al.* [54] recently reported that the bioaccessibility of Urolithin A was four times higher compared to that of the free compound. The bioaccessibility of spirulina phenolics is around 1.5 times increased by liposomal encapsulation [55].

The difference in the bioaccessibility of phenolic compounds in PE could be associated with their structures. For example, Li *et al*. [56] stated that the average bioaccessibility of phenolic acids was higher than that of flavonoids. Among hydroxybenzoic phenolic acids, the bioaccessibility was reported to decrease with an increase in the number of hydroxyl substitutions. In all our samples, the BI of caffeic acid was also lower than that of *p*-coumaric acid. The increase in the concentration of phenolic acids by digestion could be associated with the cleavage of higher molecular structures to free phenolic acids, for example, the main hydroxycinnamic acids found in the human diet, *p*-coumaric, caffeic, *trans*-cinnamic, and ferulic acids, usually as glycosides or esters of quinic acid [57]. Similarly, the possible enzymatic action on CAPE may increase the level of caffeic acids in the intestinal fluid. Although we could not determine it due to the lack of external standards, the presence of high levels of pinobanksin esters such as pinobanksin 3-acetate has been previously reported in propolis [58], and therefore the enzymes used in digestion fluids could also hydrolyze the structure and release the pinobanksin.

## Conclusions

The COVID-19 pandemic has renewed global interest in food-derived bioactive compounds, particularly propolis products, to help avoid and alleviate the symptoms of diseases. Propolis is a resinous, water-insoluble material with low bioavailability that is generally sold as solubilized in ethanol and alcohol derivative extraction solvents (*e.g.*, propylene glycol and glycerin), which limits its use in food and other consumer products. Therefore, an aqueous-based delivery system with a high loading capacity of propolis consisting of only food additives and no additional organic solvents to increase the bioaccessibility of phenolics would have potential industrial applications. In this study, we load propolis extract into liposome vesicles composed of soybean lecithin. Two food additives used as emulsifiers, polyoxyethylene sorbitan monooleate (Tween 80) and, for the first time in the literature, ammonium phosphatide, were employed in liposome formulations to enhance the loading capacity of PE. Compared to AMP, Tween 80 enabled the loading of a higher amount of PE (up to 4 %, w/v) in liposomes. Both PE loading and the employment of additional emulsifiers altered the mean particle size and surface charge of liposomes. All liposome formulations achieved an encapsulation efficiency of more than 65 % for the initially loaded PE. A total of 15 individual phenolic compounds were quantified in PE. After the samples were subjected to *in vitro* digestion, the recovery of phenolic compounds for free PE ranged from 0.03 to 5.63 %, while for liposomal propolis incorporated with Tween 80, it ranged from 4.85 to 169.46 %, and for liposomal propolis incorporated with AMP, it ranged from 6.50 to 495 %. In addition to the enhanced solubility of PE, the liposome encapsulation provided protection to the phenolic compounds of PE against the digestive fluids. In future studies, the detailed structural changes provided by AMP to liposomes and the therapeutic potential of liposomal propolis should be studied in different cell models.

## List of abbreviations

ADI: Acceptable daily intake
AMP: Ammonium phosphatide
ANOVA: Analysis of variance
ANS: Food additives and nutrient sources
BI: Bioaccessibility index
CAPE: Caffeic acid phenethyl ester
DLS: Dynamic light scattering
EE: Encapsulation efficiency, %
EFSA: European food safety authority
EU: European Union
FC: Folin-Ciocalteu
PDI: Polydispersity index
PE: Propolis extract
SGF: Simulated gastric fluid
SIF: Simulated intestinal fluid
SSF: Simulated salivary fluid
T80: Tween 80 (Polyoxyethylene sorbitan monooleate)
TEM: Transmission electron microscopy
TPC: Total phenolic content
